# Nano fuzzy alarming system for blood transfusion requirement detection in cancer using deep learning

**DOI:** 10.1038/s41598-024-66607-8

**Published:** 2024-07-10

**Authors:** Nasibeh Rady Raz, Ali Arash Anoushirvani, Neda Rahimian, Maryam Ghoerishi, Nazanin Alibeik, Masoumeh Sajadi Rad

**Affiliations:** 1 Department of Artificial Intelligence in Medical Sciences, Faculty of Advanced Technologies in Medicine, Iran University of Medical Sciences, Tehran, Iran; 2 Department of Internal Medicine, Firoozgar General Hospital, School of Medicine, Iran University of Medical Sciences, Tehran, Iran; 3 Department of Radiation Oncology, Firoozgar General Hospital, School of Medicine, Iran University of Medical Sciences, Tehran, Iran

**Keywords:** Artificial intelligence, Cancer, Blood transfusion, Deep learning, Fuzzy system, Nanonetworks, Cancer, Computational biology and bioinformatics, Oncology

## Abstract

Periodic blood transfusion is a need in cancer patients in which the disease process as well as the chemotherapy can disrupt the natural production of blood cells. However, there are concerns about blood transfusion side effects, the cost, and the availability of donated blood. Therefore, predicting the timely requirement for blood transfusion considering patient variability is a need, and here for the first-time deal with this issue in blood cancer using in vivo data. First, a data set of 98 samples of blood cancer patients including 61 features of demographic, clinical, and laboratory data are collected. After performing multivariate analysis and the approval of an expert, effective parameters are derived. Then using a deep recurrent neural network, a system is presented to predict a need for packed red blood cell transfusion. Here, we use a Long Short-Term Memory (LSTM) neural network for modeling and the cross-validation technique with 5 layers for validation of the model along with comparing the result with networking and non-networking machine learning algorithms including bidirectional LSTM, AdaBoost, bagging decision tree based, bagging KNeighbors, and Multi-Layer Perceptron (MLP). Results show the LSTM outperforms the other methods. Then, using the swarm of fuzzy bioinspired nanomachines and the most effective parameters of Hgb, PaO_2_, and pH, we propose a feasibility study on nano fuzzy alarming system (NFABT) for blood transfusion requirements. Alarming decisions using the Internet of Things (IoT) gateway are delivered to the physician for performing medical actions. Also, NFABT is considered a real-time non-invasive AI-based hemoglobin monitoring and alarming method. Results show the merits of the proposed method.

## Introduction

Blood cancer is one of the modern life concerns in a variety of ages. In such patients, blood transfusion is a requirement^1^, since the disease process as well as the chemotherapy drugs can disrupt the natural production of blood cells. Many chemotherapy drugs can temporarily disrupt the production of blood cells in the bone marrow and reduce the function of the immune system^2^. Also, patients with stem cell transplant therapy, who receive high doses of chemotherapy, have faced normal blood cell supply depletion. Therefore, a timely and accurate diagnosis of the need for blood transfusion is very important.

However, blood transfusions have side effects, and the cost, and availability of donated blood should be considered. For example, research has been done in the field of blood transfusions and immunosuppression in patients, that shows the recurrence and progress of cancer after blood transfusions^3^. In addition, soluble factors in packed red blood cells, platelets, and fresh frozen plasma (FFP) can directly stimulate tumor growth and spread^4^. Moreover, the relationship between blood transfusion and persistent inflammation, immunosuppression, and catabolism syndrome (PIICS) has been investigated in Ref.^5^, and based on that physicians should consider the risk of PIICS when performing blood transfusions in intensive care unit (ICU) patients.

Currently, blood transfusion policies usually depend on the patient's condition, the physician's individual training, skills and experience, and medical standards. Therefore, it is mandatory and a great help to provide an artificial intelligence-based assistant system to predict the need for blood transfusion at the right time for treatment. Also, the predictive systems for blood transfusions and collecting their information is a step toward intelligent world management of blood resources.

Toward this aim, nano, micro, and macro intelligent systems can accompany improving the situation by performing delivery or online monitoring and alarming. For example, in the case of robust delivery inside the body, in Ref.^6^ magnetic-field gradients are performed for anchoring and guided propulsion collectively using artificial microtubules. Also, an online monitoring and oxygen delivery system using a swarm of nanomachines is discussed in Ref.^7^ for avoiding oxygen deficiency cases.

So far, limited studies have been conducted in this field for various diseases, and the present study for the first-time deals with this issue in blood cancer using in vivo data. Previously, regression models have been used for blood transfusions. However, to determine the variable contribution and consider their interrelation, there is a need for a more intelligent method. In this paper, we use a deep recurrent neural network that uses its data from Electronic Health Records (EHRs) including demographics, vital signs, and laboratory variables. Here, in the first phase, a data set includes (N = 98) samples of cancer patients including 61 features of demographic, clinical, and laboratory data, which are selected after performing multivariate analysis and with the approval of an expert for daily forecasting. A deep recurrent long short-term memory (LSTM) neural network is then modeled. Selected features are used as input nodes of LSTM to predict the daily need for packed red blood cell transfusion. To validate the model, the cross-validation technique with 5 layers is used. Then, the results of the model are compared with network and non-network machine learning techniques. In the second phase, we use the most effective parameters which are extracted from the first phase to be the inputs for the swarm of fuzzy bioinspired nanomachines and perform a feasibility study on proposing nano fuzzy alarming (NFABT) system for blood transfusion requirement. This distributed intelligent nano fuzzy scale system performs the transfusion requirement estimation and alarms the physician for performing medical actions.

In short, the paper has the following novel aspects:

1. Data analysis for finding possible artificial intelligence-based biomarkers for blood transfusion requirements in cancer.
2. Screening blood transfusion requirement in cancer patients using a recurrent deep learning neural network and demographic, clinical, and laboratory in vivo data.
3. Early detecting of blood transfusion requirement by a collaborative nano fuzzy swarm of nanomachines.
4. Non-invasive hemoglobin measurement method for continuous transfusion requirement monitoring.

The paper has five sections. In “Literature review”, we review relevant research works. “The proposed method” describes the proposed method. Experimental results and analysis are presented in “Experimental results and analysis”. Finally, conclusions are explained in “Conclusions and future work”.

## Literature review

In cancer patients, periodic blood transfusion is one of the needs for the patients while bringing challenges. Therefore, providing a system for timely predicting the need for blood transfusion in patients is a great help to the medical staff and optimal blood management. So far, limited research has been done in predicting blood transfusion. In this section, we first review blood transfusion needs in cancer and its side effects. Then, we review blood transfusion prediction cases.

### Blood transfusion need in cancer and its side effects

Cancer patients, either due to the nature of the cancer or the treatment, may need blood transfusion. The need for blood transfusion in cancer is discussed in Ref.^1^ due to anemia and thrombocytopenia. For example, most patients with colorectal cancer (CRC) have anemia. Therefore, blood transfusion is mostly prescribed for them. Also, many chemotherapy drugs disrupt the blood cell production in the bone marrow^2^. Hence, a timely blood transfusion is of great importance.

However, the great body of literature mentioned the harmful effect of transfusion immunomodulation on the patient’s immune function. They suggest patients with cancer may have an impaired immune system^5^ and ‌blood transfusions lead to the deterioration of the natural process of the immune system in cancer cells elimination. In Ref.^3^, the effect of blood transfusion is investigated on the recurrence and mortality of CRC. Also, the variability in blood donor features including age, gender, race, and component manufacturing affect transfused products in pro- and anti-inflammatory molecules^8^. In Ref.^9^ it is suggested that blood transfusion is a personalized medicine and not a routine hospital procedure due to its negative impacts on the immune system and tumor growth stimulation.

Moreover, in Ref.^10^, the relationship between perioperative allogeneic blood transfusions and recurrence of renal cell carcinoma is investigated using multivariable proportional regression models. Also, in Ref.^11^ it is mentioned that many factors in addition to Hb are related to tumor hypoxia, and therefore it is oversimplified to consider this complex multifactorial issue as a single-factor case that can be overcome with blood transfusion. Furthermore, in Ref.^12^ risk of recurrence of colorectal cancer related to perioperative blood transfusion is discussed. They investigate laboratory, animal, and clinical evidence of inflammatory effects of blood transfusion for recurrence in the cancer surgical patient. Also, in Ref.^13^ it is discussed that there is a possible relation between transfusion ‘dose’ and cancer recurrence in cancer patients. Furthermore, the findings in Ref.^14^ suggested that blood transfusions during antitumor immunotherapy treatment has a negative impact on response rates. Hence there should be restrictive transfusion management in those patients.

In Ref.^15^ the usage of blood transfusion guidelines and targeted interventions are investigated and it is stated that by using them inappropriate transfusion is decreased. Finally, in Ref.^16^ by considering guidelines, the effect of blood transfusion and relevant risks including adverse neurodevelopmental outcomes, the difference in donor sex, and inflammation are investigated in neonates. Therefore, blood transfusion is needed in cancer while it has side effects and we should be careful during its usage. Hence, it is necessary to develop prediction models for timely transfusion.

### Blood transfusion prediction

In this section, we review several transfusion prediction models. Predicting the need for blood transfusion in cardiothoracic surgery (CT) has been presented in the research^17^ to control blood resources and a suitable indicator to assess the risk of bleeding before surgery using random forests. For surgical patients, neural networks and regression models have been used to predict the need for blood transfusion after surgery. The detection of patients who require blood transfusions^18^ leads to better operation by better preparation during and after the operation.

For the case of surgical patients who need blood transfusions during and after surgery, the prediction of the need becomes even more important. In such a case, blood transfusion is a part of therapy, and also leads to mortality reduction and quick improvement. However, those patients who receive blood transfusions are generally, at risk of blood-borne infections, ABO incompatibility, hemolytic reactions, overload transfusion-related circulatory limitations, transfusion-related acute lung injury, immunosuppression, increased risk of intraoperative infection, and early recurrence of malignancies after resection. Therefore, pre-knowledge for blood transfusion not only controls blood resources and warns of post-operative complications, but also improves the outcome of surgery and decreases cost by avoiding unnecessary blood transfusions.

Moreover, in Ref.^21^ prediction models are used to continuously measure physiological data rather than laboratory tests for hemorrhage early detection and the need for blood transfusion in the surgical ICU.

It is also stated in Ref.^19^ that due to the blood donors reduction, the optimal management of blood has become one of the important challenges of global public health, which needs an urgent solution. Blood transfusion after surgery is one of the requirements. Blood transfusion improves symptoms of ischemia and hypoxia in patients and reduces mortality, although it may also cause adverse transfusion reactions. Of course, not all patients need blood transfusions after surgery.

Currently, pre-operative testing often includes blood group determination and antibody screening (T&S), and the need for post-operative injection is not investigated. In research^19^, a blood transfusion prediction model is presented to identify high-risk patients before surgery who need a blood transfusion after surgery using a nomogram model to classify the risk of patients undergoing surgery.

One of the main issues in the health system is to provide fresh blood under proper policymaking. The study presented in Ref.^20^ is to forecast the demand for different blood groups using autoregression integrated moving average, artificial neural network, and hybrid approach using in-vivo data of 12 months. Also, the mean square error and mean absolute error measures were used as validation strategies.

Blood loss is a common issue in Endoprosthetic surgery that needs on-time blood transfusion. In the research^21^ risk factors for both blood loss and the need for blood transfusion are discussed for personalized blood transfusion calculation. Also, in Ref.^22^ a method based on fitting smooth spline functions is presented to measure total hemoglobin (SpHb) over a time window and a regression model to predict the true hemoglobin value for the end of the time window.

Moreover, in Ref.^23^ distributed machine learning (ML) as a parallel computing method is presented that can be used for distributed estimation of hemoglobin. Finally, in Ref.^22^ it is discussed that due to the variability of patient condition for anemia management, dose optimization is a challenging issue and a prediction strategy is a solution. Therefore, they use a recurrent neural network for patient history modeling and hemoglobin prediction in a cohort.

## The proposed method

In this section, we explain the data set, data preprocessing methods, blood transfusion prediction, and the proposed nano fuzzy alarming system for blood transfusion requirements.

### Dataset

We use a dataset of 98 samples of blood cancer patients including 61 features of demographic, clinical, and laboratory data from Firozgar Hospital. Since the research did not impact clinical care and all information was deidentified, the requirement for individual patient consent was waived by review boards of the Iran University of Medical Sciences (IUMS). All procedures were performed in accordance with relevant guidelines. All experimental protocols were approved by the review boards of IUMS. The data has been de-identified and is not subject to HIPAA Privacy Rule restrictions. The used features which were selected after performing multivariate analysis and approval of an expert are presented in Table 1 in three categories including demographic information, clinical features, and three sets of laboratory data.

**Table 1:** Selected features for predicting blood transfusions.

Demographic Information	N	
Male/female		
Male	55	
Female	43	
Age		
88–90	2	
74–88	14	
60–74	24	
46–60	23	
32–46	23	
18–32	12	
Cancer type		
ALL	22	
AML	27	
CLL	33	
CML	16	
History of disease	No	Yes
Diabetic states	71	27
Heart disease states	74	24
Respiratory disease	84	14
Chronic kidney disease	86	12
Smoking	65	33
Alcohol consumption	82	16
Chemotherapy	32	66
Sepsis	88	10
Active infectious	75	23
Fever	81	17
Antibiotic injection	34	64
Blood transfusion	43	55
Presence of active bleeding	95	3
Outcome		
Packed red blood cells		
Clinical features	Mean	Std Dev
Systolic blood pressure	124.64	21.64
Diastolic blood pressure	75.74	11.76
Heart rate (beats per minute)	77.13	14.46
LVEF	50.82	5.26
Laboratory data	Mean	Std Dev
Base excess	2.26	4.69
Total carbon dioxide	39.95	6.92
pH	7.35	0.06
Arterial pressure of oxygen	34.54	7.25
Oxygen saturation (pulse oximetry)	93.72	3.14
Hemoglobin	8.93	2.50
Hematocrit	28.93	7.58
Mean corpuscular hemoglobin	29.82	2.87
Mean corpuscular hemoglobin concentration	33.37	2.749
Mean corpuscular volume	86.41	7.159
Red blood cell distribution width	17.51	1.61
Platelet	108.88	67.66
Na	138.62	5.82
K	4.072	0.681
Mg	1.94	0.26
Phosphate	4.25	0.75
Calcium	8.45	0.82
Bicarbonate	22.23	4.19
Ferritin	124.42	19.08
PT	14.13	2.06
PTT	35.03	9.02
BUN	30.97	16.09
Creatinine	1.196	0.58
ALT	60.19	22.22
AST	59.58	27.29
ALP	466.31	151.93
Albumin	3.262	0.48
Bilirubin total	1.134	0.562
Bilirubin direct	0.59	0.29
Creatine kinase	134.23	132.098
Creatine kinase-MB	26.63	17.67
Lactate dehydrogenase	828.19	510.078
Troponin T	0.031	0.171

### Data preprocessing

Here we use vitals and laboratory values for each patient. We exclude values with more than 90% missingness including BMI, chemotherapy intervals, chloride, anion gap, total iron, iron-binding capacity, lactate, and transferrin. For those values with more than 50% missingness, we consider 0 as an indicator for missingness and 1 as those which are present. Then we use the median imputation with those values that have a missingness indicator. To have a similar protocol across patients in the values of heart rate, systolic blood pressure, and diastolic blood pressure, we fix the first timepoint of each recorded data at the first recording. We then normalize the continuous data before training to avoid the effect of much larger values to the rest of the variables.

We consider 1 as representative of a man and 2 as a woman in gender features. For the history of disease including diabetes, heart, respiratory, and Chronic kidney in addition to sepsis, active infection, presence of active bleeding, and fever, we use 1 as an indicator of having the disease and 0 as not having the disease. We consider 1–4 for four types of blood cancer including Acute Lymphoblastic Leukemia (ALL), Acute Myeloid Leukemia (AML), Chronic Lymphocytic Leukemia (CLL), and Chronic Myelogenous Leukemia (CML). Moreover, for the smoking and alcohol consumption in addition to chemotherapy, antibiotic injection, and blood transfusion, we use 1 as an indicator of applying and 0 as not applying. Finally, we use 0 for negative and 1 for positive Troponin T values. In the training set 7%, and in the test set 4% are labeled as receiving a packed red blood cell.

**Figure 1:**
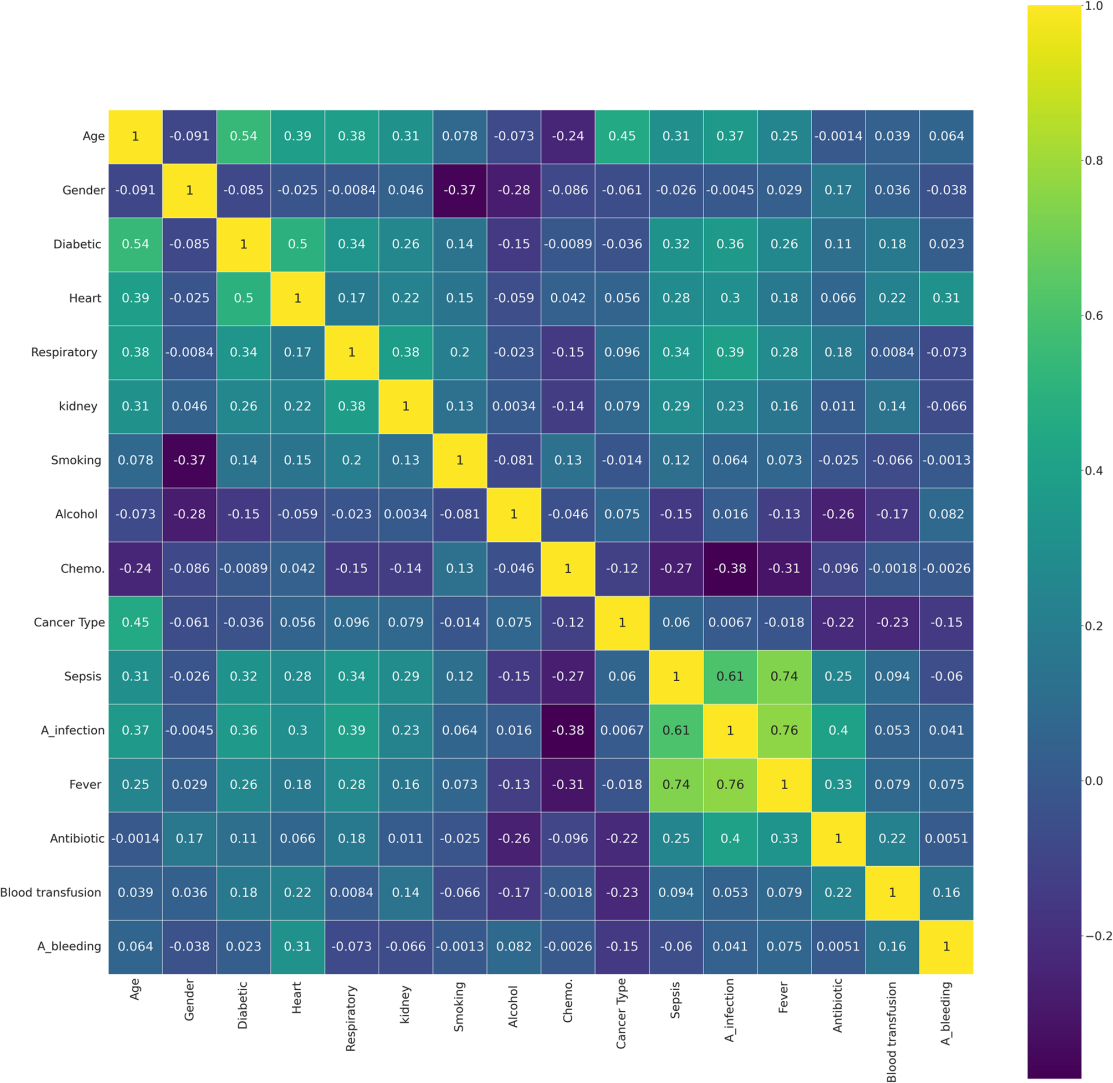
Pearson’s correlation matrix for geographical data.

**Figure 2:**
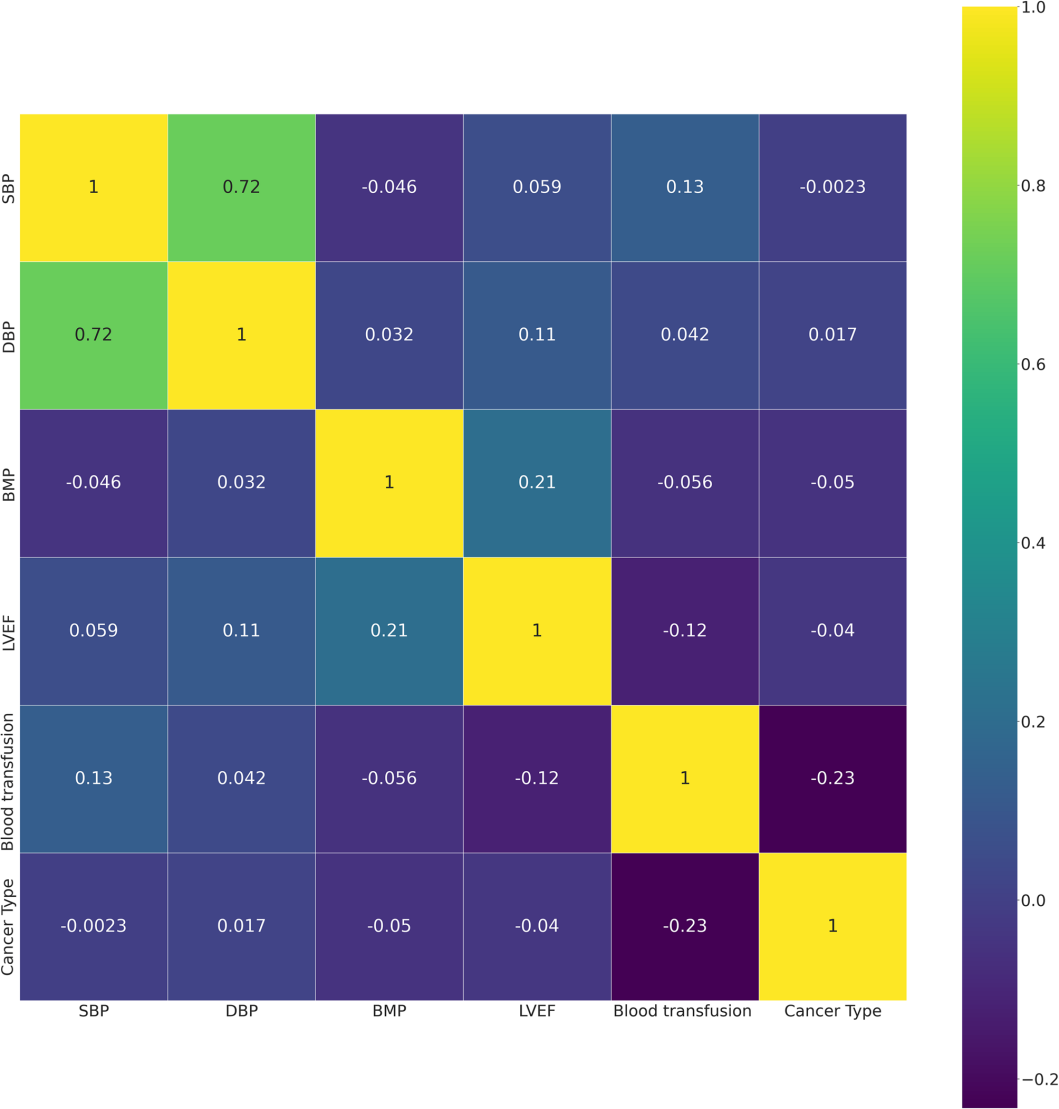
Pearson’s correlation matrix for clinical data.

**Figure 3:**
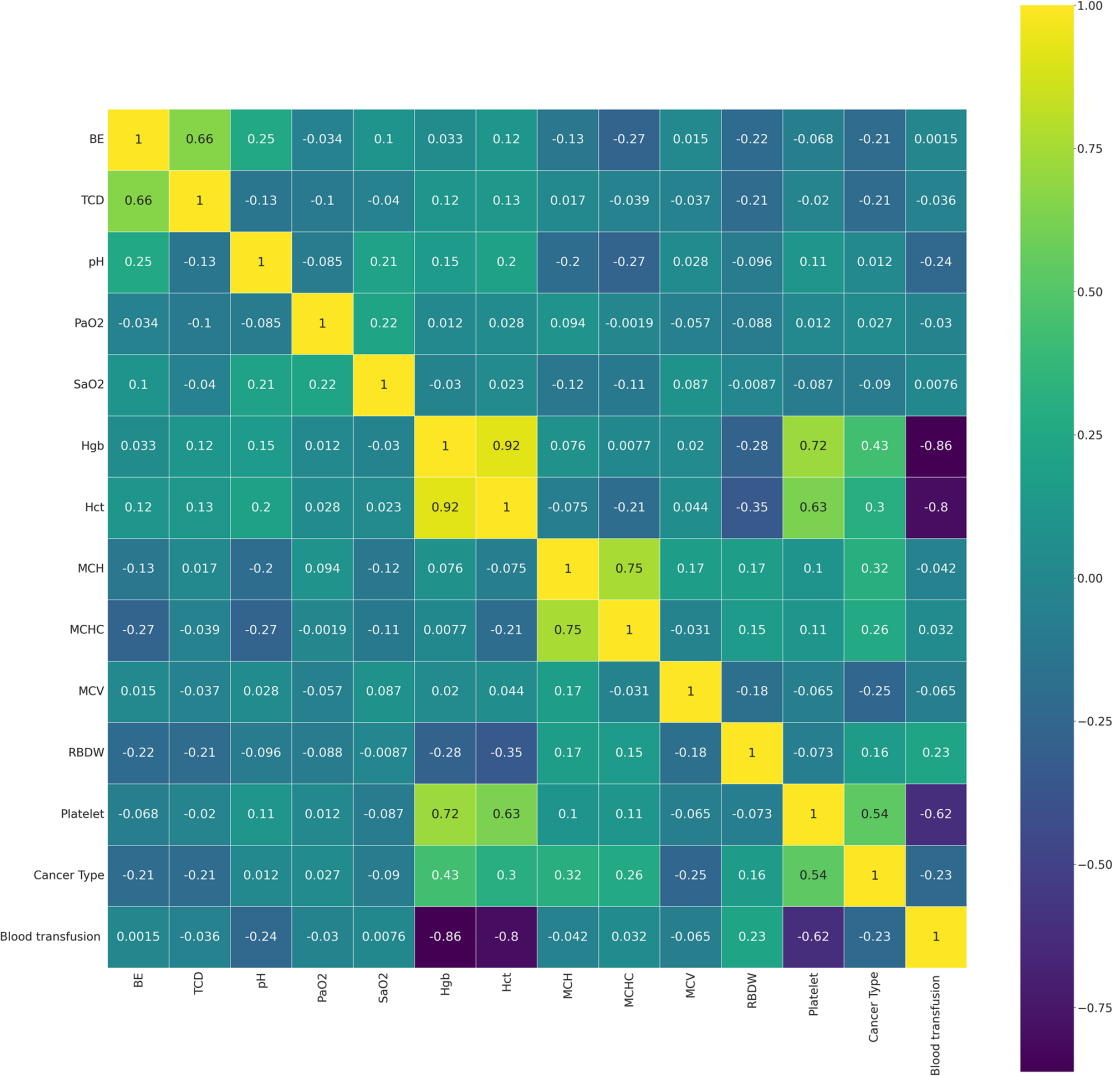
Pearson’s correlation matrix for the first set of laboratory data.

**Figure 4:**
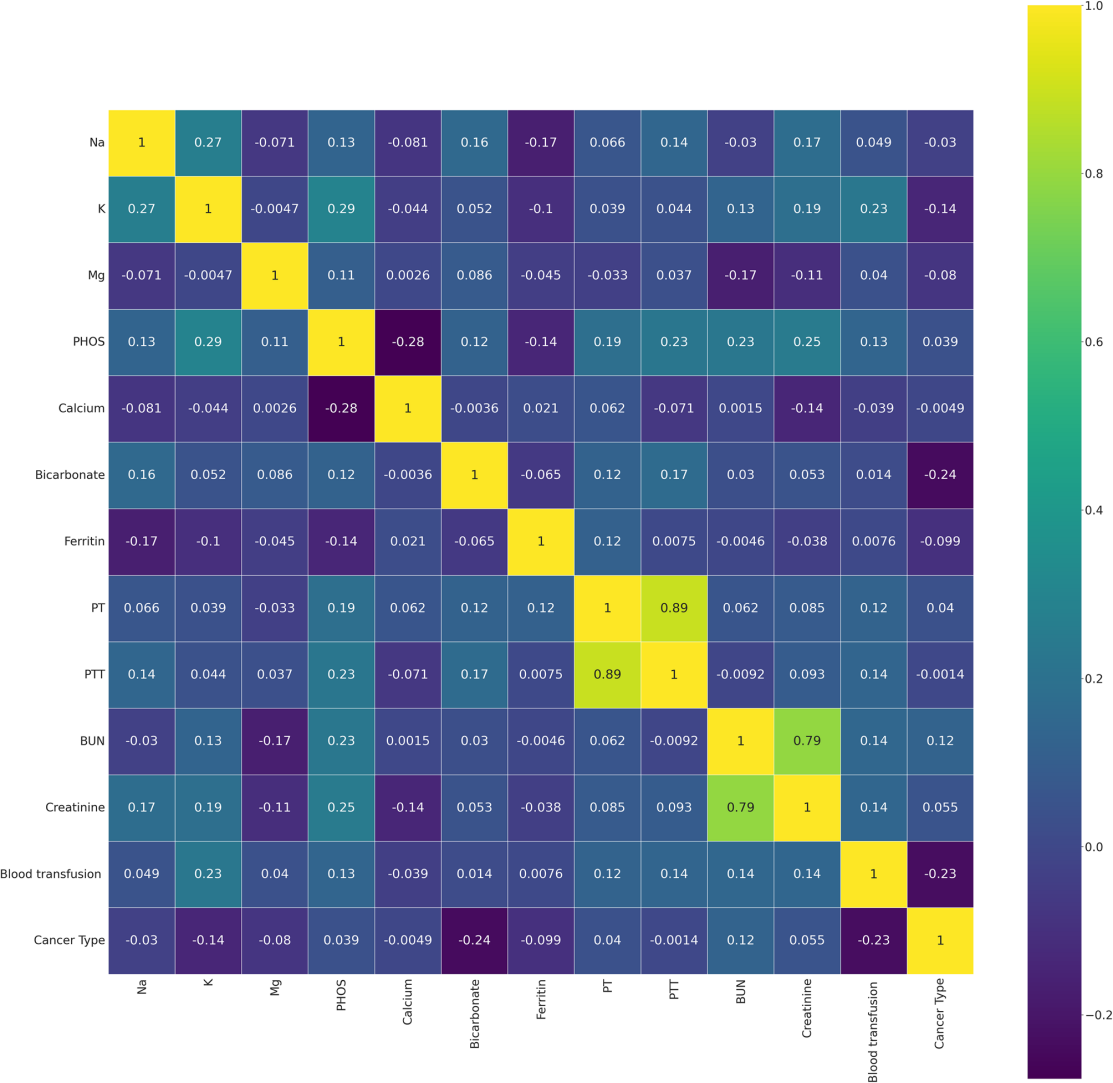
Pearson’s correlation matrix for the second set of laboratory data.

**Figure 5:**
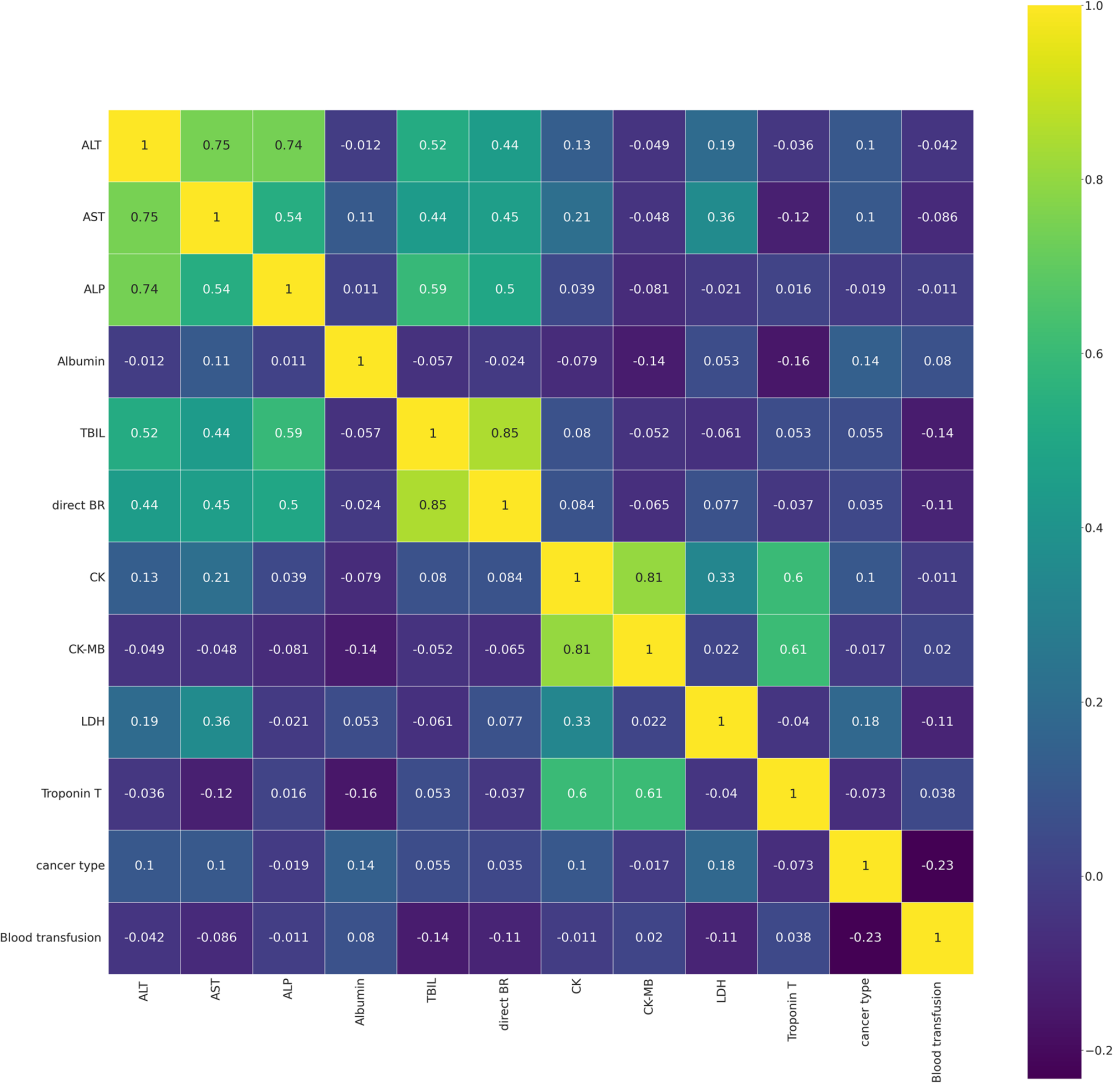
Pearson’s correlation matrix for third sets of laboratory data.

To decrease the effect of correlated variables in the neural network variables, we use a Pearson’s correlation matrix as shown in Fig. 1 for geographical data, Fig. 2 for clinical data, Figs. 3, 4, and 5 for laboratory data to validate that input variables are relatively uncorrelated. As the result shows they are uncorrelated. In Fig. 1, all demographic data except age are Boolean variables, and the rest of the variables are continuous real values.

We then detect the relevant variables. In Fig. 1 sepsis, active infection, and fever are related. DBP and SBP are related in Fig. 2. In Fig. 3 Hgb and Hct are related to platelet. Also, MCH is related to MCHC, and TCD is related to BE. In Fig. 4, BUN and creatinine are relevant. Also, PTT and PT are relevant. In Fig. 5, CK and CK-MB are relevant to Troponin T, CK is related to CK-MB, direct BR is related to TBIL, and finally, ALP and AST are related to ALT.

### Distinguishable parameters selection in blood transfusion

**Figure 6:**
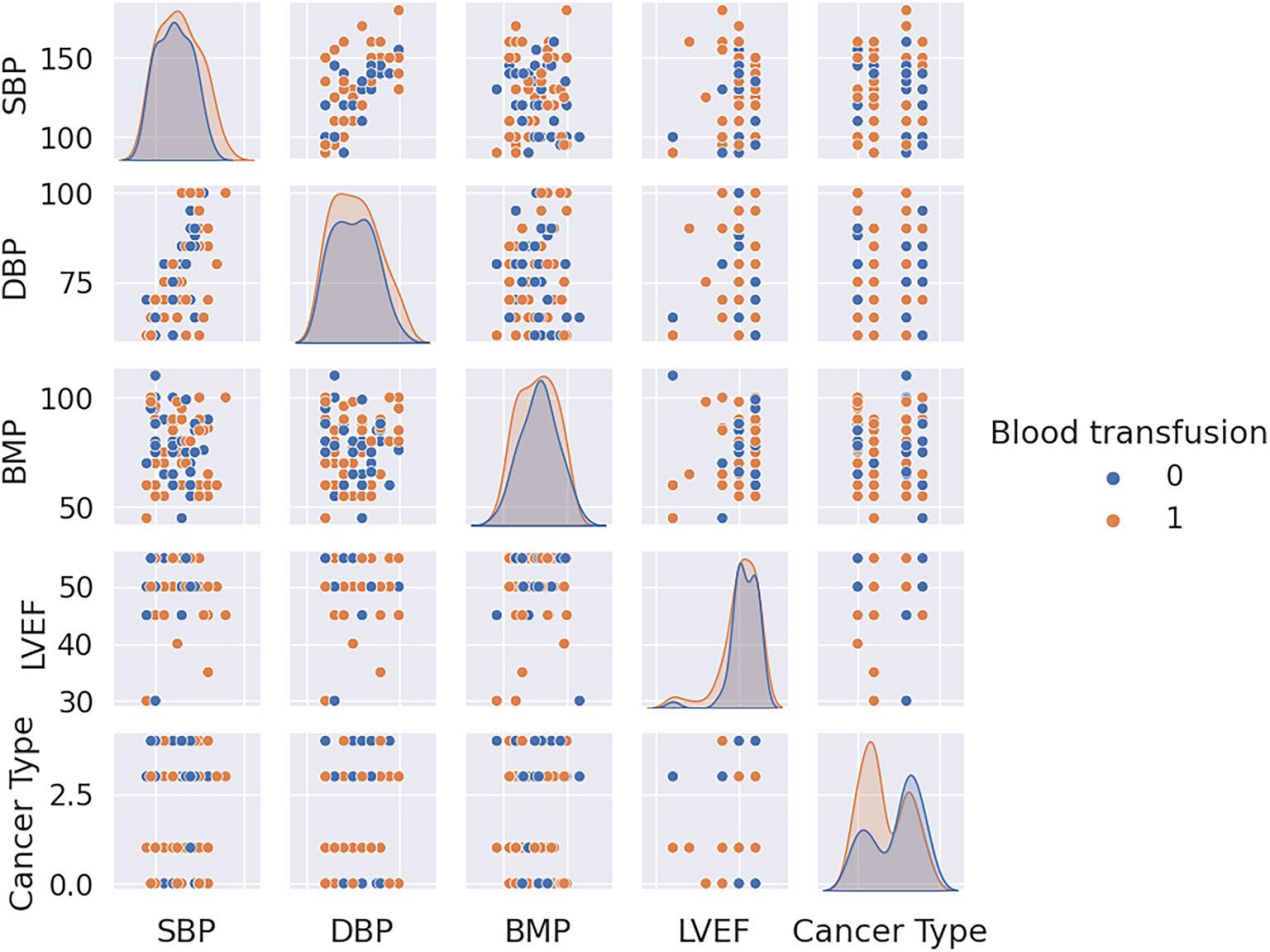
Scatterplot of clinical data for the two classes of blood transfusion.

**Figure 7:**
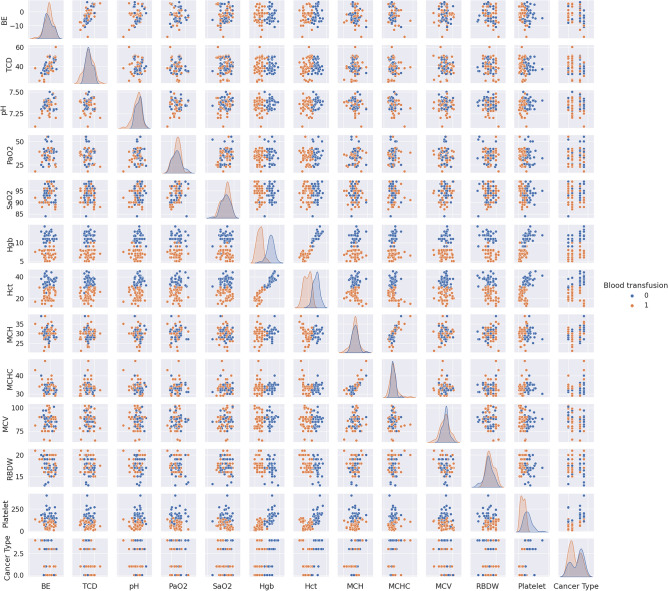
Scatterplot of laboratory data for the two classes of blood transfusion.

**Figure 8:**
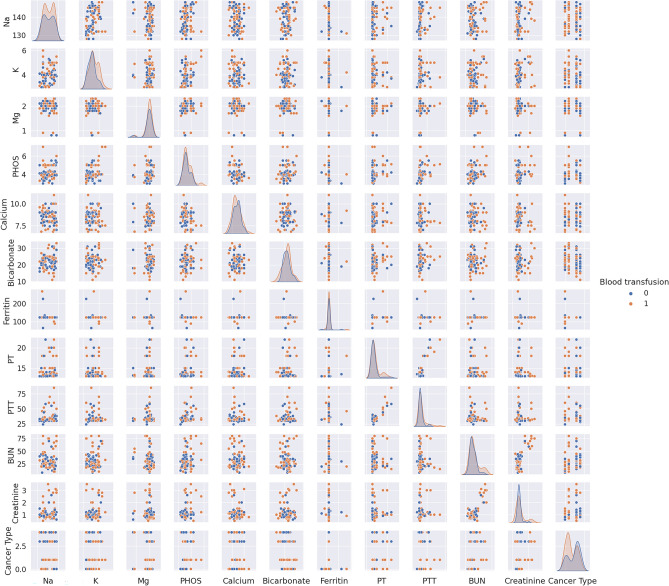
Scatterplot of second sets of laboratory data for the two classes of blood transfusion.

**Figure 9:**
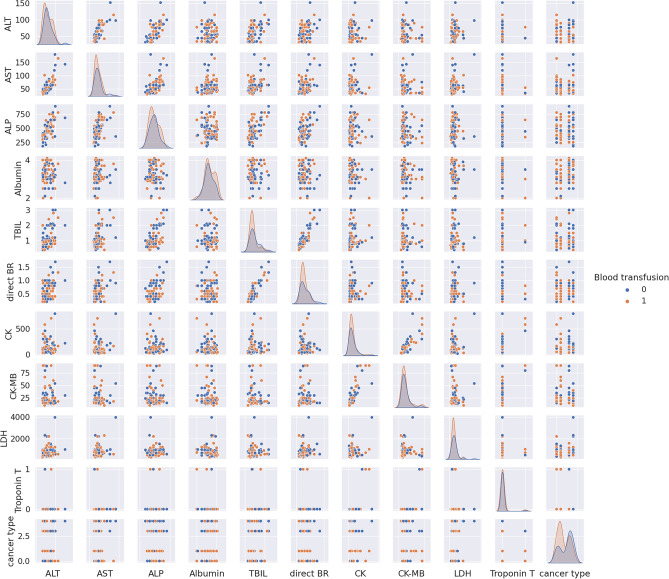
Scatterplot of third sets of laboratory data for the two classes of blood transfusion.

We first analyze data to find the distinguishable parameters in blood transfusion requirements. Hence, we illustrate the scatterplot of data depending on two categories of blood transfusion and not blood transfusion in Figs. 6, 7, 8, and 9. Accordingly, SBP in Fig. 6 and platelet, Hct, Hgb, and the variables of SpO2, PaO2, pH, TCD, and BE in the presence of Hct and Hgb in Fig. 7 are selected as the most distinguishable parameters. Furthermore, in Fig. 8, creatinine, BUN, and PTT are selected, and finally, in Fig. 9 there are no distinguishable variables.

Considering the analyzed data, we then use these distinguishable variables as a possible artificial intelligence (AI)-based biomarker for blood transfusion requirements. Hence, in this paper, we sense the changes in the values of these biomarkers using nanomachine to determine the right time for blood transfusion. For this, we first perform the prediction and then utilize the data for proposing a nano-alarming system for blood transfusion requirements.

### Blood transfusion prediction

To perform the prediction, we use a type of Recurrent Neural Network (RNN) as Long Short Term Memory (LSTM). LSTM can handle information that is passed between subsequent time iterations. In this modeling, $x\left(0\right)$, $x\left(1\right)$, …, $x\left(T-1\right)$ where represents the input variables at the beginning of each 24 h and $\widehat{y}\left(0\right)$, $\widehat{y}\left(1\right)$,…,$\widehat{y}\left(T\right)$, where $\widehat{y}\left(T\right)\in \left[0\text{,}1\right]$ is the output that predicts the blood transfusion. We consider LogSoftmax for the last layer to get the log-probabilities of the output including ${\text{p}}\left({1}\right)\text{, p}\left({2}\right)\text{, . . . , p}\left({\text{T}}\right)$ in which $p\left(t\right)$ is the log-probability of $\widehat{y}$ for the two classes.

### Nano-alarming system for blood transfusion requirement

**Figure 10:**
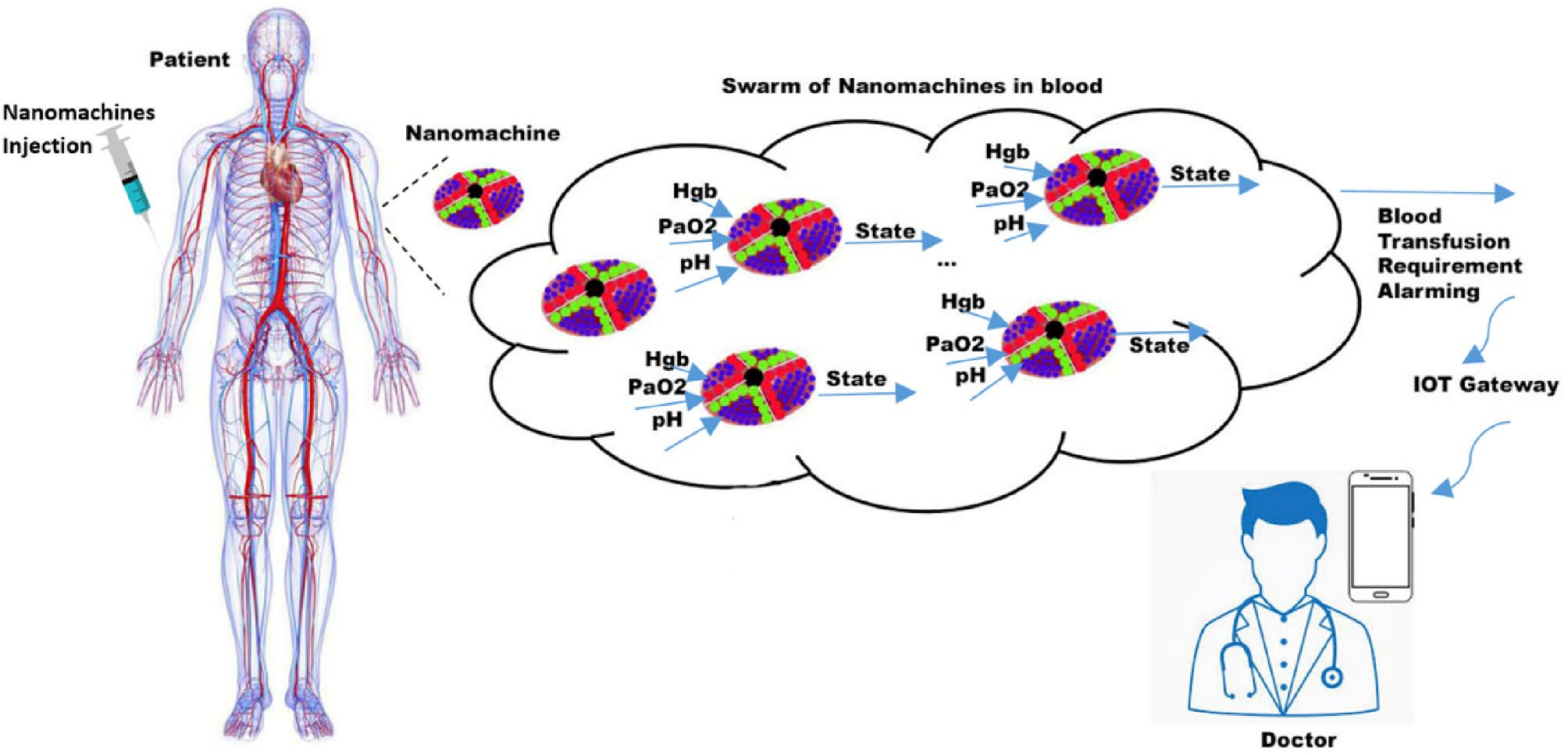
Proposed nano fuzzy alarming system (NFABT) for blood transfusion requirement.

In this section, we propose a feasibility study on nano fuzzy alarming system (NFABT) for blood transfusion requirement. Previously, inspired by red blood cells^24^ and cancer cell scaffold^25^, we proposed bio-inspired nanomachines for cancer drug delivery^26^, and oxygen deficiency cases^7^. Here, utilizing red blood cell-inspired nanomachines, we propose a nano-alarming system for blood transfusion. For this, each nanomachine is equipped with fuzzy basis functions (FBFs), and the overall fuzzy system is calculated using a swarm of FBFs. Figure 10 shows the proposed NFABT method.

Considering the analysis in “Data preprocessing” we select three parameters of Hgb, PaO_2_, and pH value for possible inputs of FBFs in NFABT. These values are sensed using embedded bio nanosensors for blood in each nanomachine. The output of this system is the state of blood regarding the need for transfusion and the overall system alarms the transfusion requirement.

Accordingly, the inputs of Hgb, PaO_2_, and pH and the output of the blood state as the marker for blood transfusion requirement in the universe of discourse of [7,16.6], [40,80], [6.3,7.8], and [0,1], respectively. Considering experts’ suggestions and clinical guidelines, we define four MFs including {Very Low, Low, Normal, high}, {Normal, Mild Hypoxemia, Moderate Hypoxemia, Severe hypoxemia}, and {Acidosis, Normal, Alkalosis} for inputs and two MFs including {Low, Normal} for output. The centers of the MFs of the inputs and outputs are {8,10.25,14.1,16.6}, {30,47.5,65.75,90}, {7.35,7.4,7.45}, and {0.25,0.75}, respectively. We also have ${4}^{2}\times {2}^{1}=32$ possible rules as presented in (1).

Particularly, we first partition the $i$th input ${z}_{j}$ to overlapping membership functions (MFs), that are assumed to be normal, complete, and consistent. Then, using the data, normal, complete, and consistent fuzzy rules are generated adaptively in the form of (1),

where rule index is ${\text{i}} \, = \, 1, 2, \ldots, {\text{R}}$, input variables are ${\text{z}}_{\text{j}} \, \left({\text{j}} \, = \, 1, 2, \ldots, {\text{m}}\right)$ and $k$ is the output variable. The ${A}_{j}^{i}$ and ${B}^{i}$ are linguistic terms that are defined by inputs and output MFs of ${\mu }_{{A}_{j}^{i}}\left({z}_{j}\right)$ and ${\mu }_{{B}^{i}}\left(k\right)$, respectively.

1 $${R}_{i}=\text{IF} {z}_{1} \,\text{is}\, {A}_{1}^{i} \,\text{and}\, {z}_{2} \,\text{is}\, {A}_{2}^{i} \,\text{and}\dots \text{and}\, {z}_{j} \,\text{is}\, {A}_{j}^{i} \,\text{then}\, k \,\text{is}\, {B}^{i},$$

We previously in Ref.^7^ proved that using triangular MFs, the sum of the multiplication of FBFs is equivalent to a common fuzzy system composed of a singleton fuzzifier, centroid defuzzifier, and product inference engine. Here, applying the proof, we use (2) for designing our fuzzy system.

2 $$f\left(z\right)=\sum_{i=1}^{R}{d}_{i}\left(z\right){\theta }_{i},$$

where ${\theta }_{i}$ is the point in the output space at which the output membership function of ${\mu }_{{B}^{i}}\left(k\right)$ reaches its maximum value of 1 and ${d}_{i}\left(z\right)$ is the FBF as defined in (3).

3 $${d}_{i}\left(z\right)=\prod_{j=1}^{m}{\mu }_{{A}_{j}^{i}}\left({z}_{j}\right),$$

where ${\mu }_{{A}_{j}^{i}}\left({z}_{j}\right)$ is input MF.

## Experimental results and analysis

We perform our algorithm using Python 3 on Intel^®^ Xeon^®^ CPU @ 2.30 GHz, RAM 12 GB, Disk 100 GB. For the blood transfusion requirement prediction, we use the data from 99 samples of blood cancer patients with 61 demographic, clinical, and laboratory features. We use LSTM for prediction although we can use other algorithms. Irregular reduction or increase of parameters in the cancer state are then considered and extracted for finding suitable AI-based biomarkers. Then, we train data for the raw data. We select a recurrent deep neural network of LSTM for performing the training. For the parameter setting, we consider 2 layers for LSTM with 128 cells and LogSoftmax for the last layer. We also use the initial learning rate of 0.01, and the maximum number of 100 epochs is used for training.

We then compare the mean prediction percent accuracies in 10 runs of network and non-network-based methods including bidirectional LSTM, Adaboost, Bagging decision tree-based, Bagging KNeighbors, and Multi-Layer Perceptron (MLP) as in Table 2. Results show the outperformance of LSTM. LSTM as a deep RNN performs well in sequence data better than classical NNs, e.g., MLP, and non-network-based methods, e.g., Adaboost.

**Table 2:** Mean prediction percent accuracy in 10 runs.

Methods	Accuracy (%)
MLP	74.3
Bagging KNeighbors	87.6
Adaboost	89.5
Bagging decision tree based	90.2
Bidirectional LSTM	92.4
LSTM	**94.2**

Better values are in bold.

We discuss the effect of input data of Hgb, PaO_2_, and pH in scenarios of finding the best input combination. As the results are shown in Table 3, the combination of Hgb and pH leads to better RMSE results. We further evaluate the robustness of the proposed system against noise. For this purpose, we add the white Gaussian noise (AWGN) with different signal-to-noise ratios (SNR) of 5, 10, and 30 dB. According to the results in Table 3, the method is robust against measurement noise.

**Table 3:** Average RMSE of the proposed NFABT in 10 runs.

Approach	RMSE	RMSE with SNR level of 30 dB	RMSE with SNR level of 10 dB	RMSE with SNR level of 5 dB
NFABT with Hgb and PaO_2_ inputs	0.2834	0.3785	0.3724	0.4158
NFABT with Hgb and pH inputs	**0.2085**	0.3778	0.3420	0.3425
NFABT with PaO_2_ and pH inputs	0.4806	0.4396	0.4382	0.4824

Better values are in bold.

The NFABT with a combination of PaO_2_ and pH inputs leads to the introduction of a non-invasive AI-based hemoglobin measurement method. For continuous monitoring e.g. in pediatrics there is a great need for such non-invasive methods. We compare NFABT with current non-invasive hemoglobin measurement methods and commercial devices, such as HemoCue^®^^27^, Masimo Pronto^®^^28^, and Digital Hemoglobinometer^29^ which are all monitoring. NFABT is a real-time AI-based monitoring and alarming method with a future therapeutic potentiality. The results are presented in Table 4, which shows the NFABT outperforms the rest.

**Table 4:** Comparing non-invasive hemoglobin estimation methods.

Approach	Case	Capability	Accuracy %
HemoCue^®^^27^	Anemia	Non-invasive monitoring, capillary/venous samples	58.6%
Masimo Pronto^®^^28^	Critically ill pediatric	Non-invasive monitoring, reduce cost of care	70.3%
Digital Hemoglobinometer^29^	Anemia	Non-invasive monitoring, easy-to-read result	83%
NFABT with PaO_2_ and pH inputs	Cancer	Non-invasive continues monitoring, alarming for clinical action,	**94.1%**

Better values are in bold.

## Discussion

Blood is a valuable biological resource and its transfusion is a need in cancer while it has its side effects. Therefore, the blood transfusion issue needs multidisciplinary, emergent, and innovative solutions. The proposed NFABT approach by presenting a blood transfusion requirement prediction and a non-invasive AI-based hemoglobin measurement method using PaO_2_ and pH inputs provides on-time blood transfusion and non-invasive hemoglobin measurement monitoring. Hence, it has impacts on health professionals and public health. Health professionals can use NFABT for blood transfusion recommendations at the point of care. Also, NFABT can offer non-invasive hemoglobin measurement methods for vulnerable patients e.g., pediatrics and the elderly. Moreover, NFABT can estimate the amount of required blood resources in health centers and alarm health professionals to be prepared for it, finally, the proposed method offers a blood management strategy for omitting unnecessary blood transfusion.

On the other hand, for public health, the proposed method by focusing on blood safety and availability, moves toward personalized blood transfusion instead of a hospital routine transfusion task. Hence, it keeps the public away from the immunologic and infectious risks of blood transfusion, protects global blood resources, and can connect to emergent blood delivery systems such as drone-based blood delivery.

## Conclusions and future work

Periodic blood transfusion is a need in cancer patients and has its challenges such as transfusion side effects, the cost, and the availability of donated blood. Hence, timely blood transfusion prediction is of great interest. Here, using in vivo data for the first time we work on this issue. Particularly, we use a data set of 98 samples of blood cancer patients including 61 features of demographic, clinical, and laboratory data. After performing multivariate analysis and approval of an expert, effective parameters for blood transfusion are selected. Then we use a Long Short-Term Memory (LSTM) neural network to predict a need for packed red blood cell transfusion and we perform the cross-validation technique for validation. We compare the result with 5 other networking and non-networking machine learning algorithms including bidirectional LSTM, AdaBoost, bagging decision tree based, bagging KNeighbors, and Multi-Layer Perceptron (MLP). Results show the LSTM outperforms the other methods. We then propose a feasibility study on the noninvasive nano fuzzy alarming (NFABT) system for blood transfusion requirements using the swarm of bioinspired nanomachines that utilize Fuzzy Basis Functions (FBFs). Accordingly, the most distinguishable parameters for blood transfusion are derived including Hgb, PaO_2_, and pH, and they are used for the inputs of the NFABT and the output of the system is the blood transfusion requirement state. Results are then delivered to the physician as an alarming decision using the Internet of Things (IoT) gateway for performing medical actions. Also, NFABT is considered a real-time non-invasive AI-based hemoglobin monitoring and alarming method. Results show the merits of the proposed method.

## Data Availability

The datasets used and/or analyzed during the current study are available from the corresponding author upon reasonable request.
